# GC-MS Discrimination of Citrulline from Ornithine and Homocitrulline from Lysine by Chemical Derivatization: Evidence of Formation of *N*^5^-Carboxy-ornithine and *N*^6^-Carboxy-lysine

**DOI:** 10.3390/molecules26082301

**Published:** 2021-04-15

**Authors:** Svetlana Baskal, Alexander Bollenbach, Dimitrios Tsikas

**Affiliations:** Core Unit Proteomics, Institute of Toxicology, Hannover Medical School, 30625 Hannover, Germany; baskal.svetlana@mh-hannover.de (S.B.); bollenbach.alex@gmail.com (A.B.)

**Keywords:** amino acids, derivatization, esterification, GC-MS, pentafluoropropionic anhydride, ureide

## Abstract

Derivatization of amino acids by 2 M HCl/CH_3_OH (60 min, 80 °C) followed by derivatization of the intermediate methyl esters with pentafluoropropionic anhydride (PFPA) in ethyl acetate (30 min, 65 °C) is a useful two-step derivatization procedure (procedure A) for their quantitative measurement in biological samples by gas chromatography-mass spectrometry (GC-MS) as methyl ester pentafluoropropionic (PFP) derivatives, (Me)_m_-(PFP)_n_. This procedure allows in situ preparation of trideutero-methyl esters PFP derivatives, (d_3_Me)_m_-(PFP)_n_, from synthetic amino acids and 2 M HCl/CD_3_OD for use as internal standards. However, procedure A converts citrulline (Cit) to ornithine (Orn) and homocitrulline (hCit) to lysine (Lys) due to the instability of their carbamide groups under the acidic conditions of the esterification step. In the present study, we investigated whether reversing the order of the two-step derivatization may allow discrimination and simultaneous analysis of these amino acids. Pentafluoropropionylation (30 min, 65 °C) and subsequent methyl esterification (30 min, 80 °C), i.e., procedure B, of Cit resulted in the formation of six open and cyclic reaction products. The most abundant product is likely to be *N*^5^-Carboxy-Orn. The second most abundant product was confirmed to be Orn. The most abundant reaction product of hCit was confirmed to be Lys, with the minor reaction product likely being *N*^6^-Carboxy-Lys. Mechanisms are proposed for the formation of the reaction products of Cit and hCit via procedure B. It is assumed that at the first derivatization step, amino acids form (*N*,*O*)-PFP derivatives including mixed anhydrides. At the second derivatization step, the Cit-(PFP)_4_ and hCit-(PFP)_4_ are esterified on their *C*^1^-Carboxylic groups and on their activated *N*^ureido^ groups. Procedure B also allows in situ preparation of (d_3_Me)_m_-(PFP)_n_ from synthetic amino acids for use as internal standards. It is demonstrated that the derivatization procedure B enables discrimination between Cit and Orn, and between hCit and Lys. The utility of procedure B to measure simultaneously these amino acids in biological samples such as plasma and urine remains to be demonstrated. Further work is required to optimize the derivatization conditions of procedure B for biological amino acids.

## 1. Introduction

**Figure 1:**
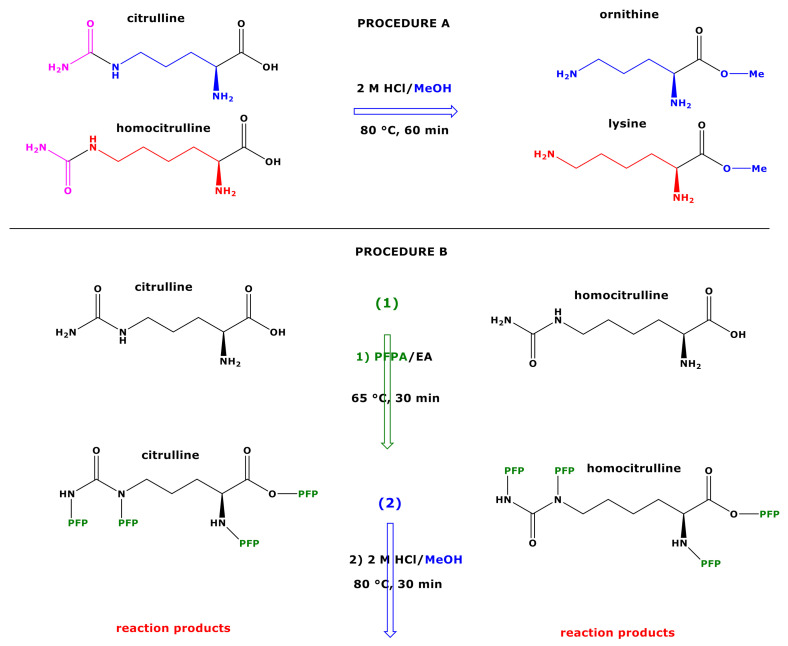
Upper panel, procedure (**A**) Schematic of the reactions of citrulline and homocitrulline with 2 M HCl/MeOH forming the methyl esters of ornithine and lysine, respectively. Lower panel, procedure (**B**) Schematic of the two-step derivatization of citrulline (left) and homocitrulline (right) first with PFPA/EA to form their PFP derivatives with the proposed formulas Cit-(PFP)_4_ and hCit-(PFP)_4_, respectively. Subsequently, these derivatives react with 2 M HCl/MeOH (procedure B) to form reaction products that were characterized structurally by GC-MS in the present study. Cit, citrulline; hCit, homocitrulline; MeOH, methanol; PFPA, pentafluoropropionic anhydride; PFP, pentafluoropropionyl residue; EA, ethyl acetate.

Analysis of amino acids, dipeptides, and tripeptides such as glutathione by gas chromatography-mass spectrometry (GC-MS) requires suitable derivatization reactions to convert them into volatile and thermally stable derivatives [1,2,3,4,5,6,7,8,9]. Derivatization of amino acids with 2 M HCl in methanol (CH_3_OH) (60 min, 80 °C) yields their mono- and di-methyl esters. Subsequent reaction with pentafluoropropionic anhydride (PFPA) in ethyl acetate (30 min, 65 °C) generates the *N*- and *O*-pentafluoropropionyl (PFP) derivatives. The methyl ester (Me) PFP derivatives ((Me)_m_-(PFP)_n_) obtained by this procedure (here designated as procedure A) are useful for the quantitative measurement of biological amino acids by GC-MS [7]. However, the carbamoyl-amino acids citrulline (Cit) and homocitrulline (hCit) (Figure 1) are converted under these reaction conditions into the methyl esters of ornithine (Orn) and lysine (Lys), respectively [7]. Analogously, glutamine (Gln) and asparagine (Asn) are converted into glutamate (Glu) and aspartate (Asp), respectively [7]. For not yet fully understood reasons, the derivatization procedure A was found to be not useful for the GC-MS analysis of *N*^G^,*N*′^G^-dimethylarginine (symmetric dimethylarginine, SDMA), in contrast to its structural isomer *N*^G^,*N*^G^-dimethylarginine (asymmetric dimethylarginine, ADMA) and to their precursor arginine. This difficulty was in part overcome by using a single derivatization reaction with PFPA, which most likely generates the *tetrakis*(pentafluoropropionyl) derivative of SDMA, i.e., SDMA-(PFP)_4_ [8]. This derivatization reaction, i.e., (*N*,*O*)-pentafluoroprionylation, enables quantitative measurement of SDMA in human urine, but requires the use of commercially available stable-isotope labelled SDMA analogue such as [*N*^G^,*N*′^G^-^2^H_6_]dimethylarginine [8] and is less sensitive compared to the GC-MS analysis of ADMA as Me-PFP derivative. Interestingly, the tripeptides glutathione and its analogue ophthalmic acid were also found to react with PFPA under the same derivatization conditions, which enabled their GC-MS analysis [9].

The aim of the present study was to find derivatization conditions that would allow discrimination of Cit from Orn, and of hCit from Lys. Our previous observations that SDMA can be measured in human urine by GC-MS by using PFPA/EA as the first derivatization step [8] prompt us to investigate whether the derivatization of Cit and hCit with PFPA/EA as the first step may also be useful for their GC-MS analysis and for their discrimination from Orn and Lys, respectively. Analogous to SDMA, we assumed intermediate formation of Cit-(PFP)_4_ and hCit-(PFP)_4_ (Figure 1). In order to investigate potential reactions of the putative intermediates, we coupled the PFPA/EA derivatization with the classical esterification with 2 M HCl/CH_3_OH and with 2 M HCl/CD_3_OD to prepare stable-isotope-labelled analogs of Cit and hCit. De facto, this resulted in a reversed order of the original two-step derivatization procedure A, which is specified as procedure B in the present work (Figure 1). In most investigations using derivatization procedure B, we used experimental conditions previously found to be optimum for the derivatization and GC-MS analysis of amino acids and the tripeptides glutathione and ophthalmic acid [7,8,9].

## 2. Materials and Methods

### 2.1. Chemicals, Materials and Reagents

All amino acids (chemical purity, 95 to 98%) were obtained from Sigma-Aldrich. Tetradeuterated methanol (CD_3_OD, 99% at ^2^H) and pentafluoropropionic anhydride were supplied by Aldrich (Steinheim, Germany). Methanol was obtained from Chemsolute (Renningen, Germany). Hydrochloric acid (37 wt%) was purchased from Baker (Deventer, The Netherlands). Ethyl acetate was obtained from Merck (Darmstadt, Germany). Glassware for GC-MS (1.5 mL autosampler glass vials and 0.2 mL microvials) and the fused-silica capillary column Optima 17 (15 m × 0.25 mm I.D., 0.25 µm film thickness) were purchased from Macherey–Nagel (Düren, Germany). Separate stock solutions of amino acids were prepared by dissolving accurately weighed amounts of commercially available amino acids in deionized water. Stock solutions were diluted with deionized water as appropriate.

For the preparation of unlabelled methyl esters and deuterium-labelled methyl esters of amino acids, two derivatization reagents were prepared. To 80 mL ice-cold CH_3_OH were added 16 mL of 37 wt% HCl slowly under gentle mixing. Analogously, to 80 mL ice-cold CD_3_OD, 16 mL of 37 wt% HCl were added slowly under gentle mixing. The concentration of HCl in these methanolic solutions was each 2 M. In the present article, these solutions are denoted as 2 M HCl/CH_3_OH and 2 M HCl/CD_3_OD, respectively. The PFPA-ethyl acetate reagent (PFPA/EA) was prepared daily by diluting pure PFPA in ethyl acetate (EA) (1:4, *v*/*v*).

### 2.2. Derivatization Procedures A and B for Amino Acids and Generation of GC-MS Spectra

*Procedure A.* Solid amino acids were derivatized first with 2 M HCl/CH_3_OH or 2 M HCl/CD_3_OD and then with PFPA/EA in autosampler glass vials. Briefly, residues were reconstituted in 100 µL aliquots of a 2 M HCl/CH_3_OH or 2 M HCl/CD_3_OD solution and the glass vials were tightly sealed. Esterification was performed by heating the samples for 60 min at 80 °C. After cooling the samples of the esterification reaction to room temperature, solvents and reagents were evaporated to dryness under a stream of nitrogen. Aliquots (100 µL) of the PFPA/EA solution were added, and the glass vials were tightly sealed and heated for 30 min at 65 °C to prepare *N*-pentafluoropropionic amides of the methyl esters. Then, residues were treated first with 200 µL aliquots of 400 mM borate buffer, pH 8.5, and immediately thereafter with 200 µL aliquots of toluene, followed by immediate vortex-mixing for 60 s and centrifugation (4000× *g*, 5 min, 18 °C). Aliquots (150 µL) of the upper organic phase were transferred into autosampler glass vials equipped with microinserts, and the samples were sealed and subjected to GC-MS analysis.

*Procedure B.* Solid amino acids were derivatized first with PFPA/EA (30 min, 65 °C) and then with 2 M HCl/CH_3_OH or 2 M HCl/CD_3_OD (30 min, 80 °C). Briefly, aliquots (100 µL) of a freshly prepared PFPA/EA solution were added, the glass vials were tightly sealed and heated for 30 min at 65 °C to prepare *N*-pentafluoropropionic amides of the methyl esters. After cooling the samples to room temperature, solvents and reagents were evaporated to dryness under a stream of nitrogen. Then, residues were reconstituted in 100 µL aliquots of a 2 M HCl/CH_3_OH or 2 M HCl/CD_3_OD solution and the glass vials were tightly sealed. Esterification was performed by heating the samples for 30 min at 80 °C. After cooling to room temperature, solvents and reagents were evaporated to dryness under a stream of nitrogen. Residues were treated directly with toluene (200 µL), shortly vortex-mixed, aliquots (150 µL) of the upper organic phase were transferred into autosampler glass vials equipped with microinserts, and the samples were sealed and subjected to GC-MS analysis.

### 2.3. Generation of GC-MS Spectra

GC-MS spectra were obtained using negative-ion chemical ionization (NICI) after separate derivatization of 5 nmol of each amino acid using both derivatization procedures as described above. The derivatives were extracted with toluene (1 mL), 1 µL aliquots containing 5 pmol of each analyte (assuming quantitative yield) were injected in the splitless mode, and mass spectra were generated in the scan mode in the mass-to-charge (*m/z*) range 50 to 650 (1 s per scan). The GC-MS software Xcalibur and Quan Browser were used. ChemDrawProfessional 15.0 was used to draw chemical structures and to convert structures into names. GraphPad Prism 7.0 (San Diego, CA, USA) was used in statistical analyses and to prepare graphs.

### 2.4. GC-MS Conditions

All analyses were performed on a GC-MS apparatus consisting of a single quadrupole mass spectrometer model ISQ, a Trace 1210 series gas chromatograph, and an AS1310 autosampler from ThermoFisher (Dreieich, Germany). The injector temperature was kept at 280 °C. Helium was used as the carrier gas at a constant flow rate of 1.0 mL/min. The oven temperature was held at 40 °C for 0.5 min and ramped to 210 °C at a rate of 15 °C/min and then to 320 °C at a rate 35 °C/min. Interface and ion-source temperatures were set to 300 °C and 250 °C, respectively. Electron energy was 70 eV and electron current 50 µA. Methane was used as the reagent gas for NICI at a constant flow rate of 2.4 mL/min. In quantitative analyses, the dwell time was 100 ms for each ion in the selected-ion monitoring (SIM) mode and the electron multiplier voltage was set to 1400 V.

## 3. Results

### 3.1. Derivatization of Citrulline and Structural Characterization of Its Reaction Products by GC-MS

Scanning of the Cit samples derivatized by procedure B resulted in the elution each of six GC-MS peaks using CH_3_OH (Supplementary Materials Figure S1A) and CD_3_OD (Figure S1B). In the latter case, the peaks I, II, V, and VI eluted a few seconds in front of the peaks of the Cit sample derivatized with CH_3_OH, indicating the presence of deuterium atoms in these peaks [7] (see Table 1). The almost identical retention times of the minor peaks III (retention time, 9.36 min) and peaks IV (retention time, 9.67 min) suggest that they are not methyl esters, but rather cyclic compounds.

The mass spectra of the peaks I (retention time, 8.36 min, 8.33 min) contained four corresponding ions that differed by 3 Da each, suggesting the presence of a single methylated carboxylic group (Figure S1(A1,B1)) (Table 1). A tentative structure of this molecule could be (*S*)-3-amino-2-oxopiperidine-1-Carboxylic acid (non-derivatized).

Derivatization of Cit by procedure B resulted in the formation of the peaks II (retention time, 8.67 min, 8.63 min) (Supplementary Materials: Figure S1(A1,B1)) (Table 1). Peaks II had virtually the same mass spectra as the unlabelled Me-PFP (d_0_Me-PFP) and the labelled Me-PFP (d_3_Me-PFP) derivatives of Orn (Figure S1(C1,C2)), indicating conversion of Cit to Orn by both procedures as observed previously using procedure A [7].

The mass spectra in combination with the retention times of the peaks III and the peaks IV suggest that the peak III corresponds to (*S*)-3-amino-4,5-dihydropyridin-2(3*H*)-one (Figure S1(A3,B3)) and peak IV corresponds to (*S*)-3-aminopiperidin-2-one (Figure S1(A4,B4)) (Table 1).

The mass spectra of the minor peaks V (retention time, 10.57 min, 10.52 min) contained corresponding ions that did not differ (*m/z* 162) or did differ by 3 Da each (*m/z*, 301/298; *m/z*, 489/486) suggesting the presence of an intact methylated carboxylic group and presumably a fragmented methyl ester (Figure S1(A5,B5)) (Table 1). A tentative structure of this molecule could be (*S*)-2-amino-5-(Carboxyamino)pentanoic acid, which could be trivially named *N*^5^-Carboxy-ornithine.

The most intense GC-MS peaks of Cit derivatized by procedure B were the peaks VI, which eluted at 10.75 min (using CH_3_OH) and 10.71 min (using CD_3_OD) (Figure S1(A6,B6)). The GC-MS spectra of these peaks contained several corresponding mass fragments that differed by 3 Da (*m/z* 298/301) or 6 Da (*m/z* 296/290, *m/z* 336/330, *m/z* 355/349) suggesting the presence of two carboxylic groups in these ions (Table 1).

### 3.2. Effects of the PFPA/EA Derivatization Time in Procedure B on the Reaction Products

The derivatization conditions used in procedure A in the present study were found to be optimal in previous studies [7,8]. In this experiment, we investigated the effect of the derivatization time of the pentafluoropropionylation reaction of Cit in procedure B. For this, two sets of 10 µL aliquots of Cit samples in distilled water (50, 100, 150, 200, 250 µM) were derivatized first with PFPA/EA at 65 °C for 30 min and subsequently with 2 M HCl/CH_3_OH for 10, 20, 30, 40, and 60 min at 80 °C. A 100 µM Cit sample in distilled water served as internal standard and was derivatized in parallel under the same conditions using 2 M HCl/CD_3_OD. After toluene extraction, GC-MS analysis was performed by SIM of *m/z* 298 and *m/z* 301 for peak I, *m/z* 418 and *m/z* 421 for peak II (i.e., Orn), *m/z* 298 and *m/z* 301 for peak V, and *m/z* 330 and *m/z* 336 for peak VI (see Table 1).

**Figure 2:**
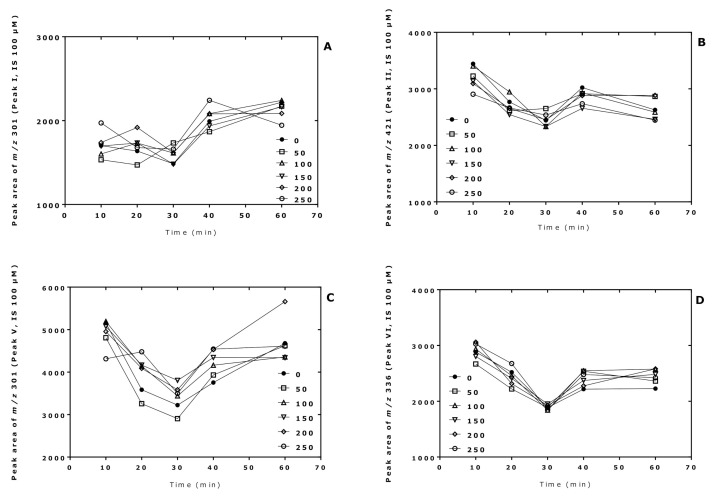
Time profiles of the peak areas of the internal standards (generated from 100 µM Cit) upon derivatization of aqueous Cit (0, 50, 100, 150, 200, 250 µM; see insert) using procedure B, i.e., first with PFPA/EA for a fixed time of 30 min at 65 °C and subsequently with 2 M HCl/CH_3_OH (2 M HCl/CD_3_OD for the internal standards) at 80 °C for the indicated times. (**A**) Peak I; (**B**) Peak II; (**C**) Peak V; (**D**) Peak VI. See also Table 1 and Figure 3.

**Figure 3:**
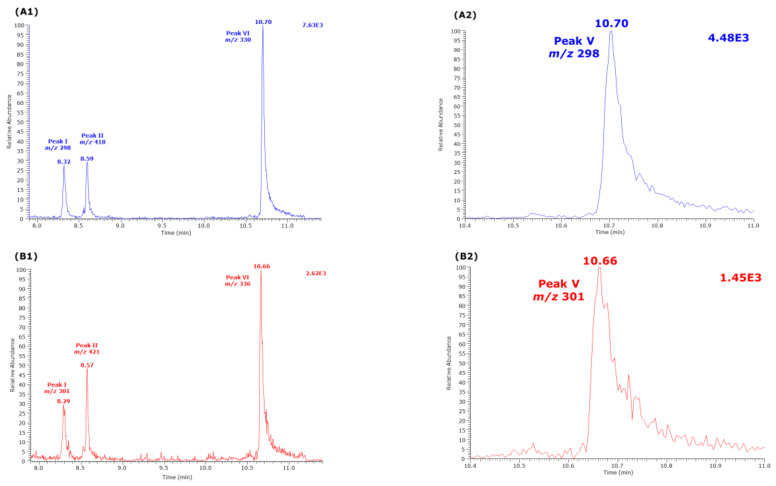
Partial GC-MS chromatograms from the analysis of an aqueous citrulline sample (250 µM) derivatized by procedure B, i.e., first with PFPA/EA (30 min, 65 °C) followed with 2 M HCl in CH_3_OH or CD_3_OD (60 min, 80 °C). Selected ion monitoring (SIM, 100 ms) of (**A1**,**A2**) *m/z* 298 and *m/z* 301 for Peak I, *m/z* 418 and *m/z* 421 for Peak II, and *m/z* 330 and *m/z* 336 for Peak VI was performed. (**B1**,**B2**) SIM (100 ms) of *m/z* 298 and *m/z* 301 for Peak V. See also Table 1 and Figure 2.

**Figure 4:**
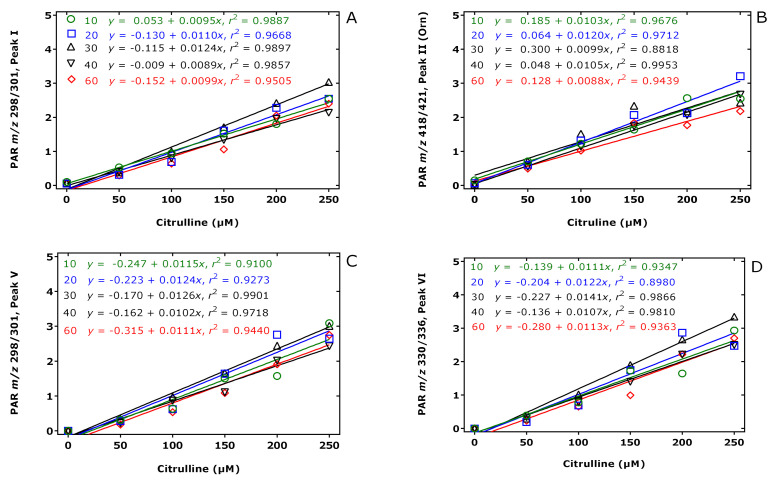
Linear regression analysis between the peak area ratio (PAR) values (*y*) for (**A**) Peak I, (**B**) Peak II, (**C**) Peak V, and (**D**) Peak VI to the corresponding internal standards (generated from 100 µM Cit) and the Cit concentration (*x*) upon derivatization of aqueous Cit (0, 50, 100, 150, 200, 250 µM) first with PFPA/EA for a fixed time of 30 min at 65 °C and subsequently with 2 M HCl/CH_3_OH (2 M HCl/CD_3_OD for the internal standard) at 80 °C for 10 (green circles), 20 (blue squares), 30 (black upper triangles), 40 (black lower triangles), 60 (red diamonds) min. Insets indicate the regression equations. Note that Peak II corresponds to the Orn derivative. See also Table 1 and Figure 3.

The results of this experiment are illustrated in Figure 2, Figure 3 and Figure 4. The peak area of the internal standards varied between 11 and 16% (*m/z* 301, Peak I), between 7 and 14% (*m/z* 421, Peak II), between 10 and 21% (*m/z* 301, Peak V), and between 13 and 17% (*m/z* 336, Peak VI). There were significant time effects for all monitored internal standards (*P* < 0.0001, two-way ANOVA) and a Cit concentration effect for Peak II (*P* = 0.043, two-way ANOVA). With the exception of the Peak I, the peak areas of the Peaks II, V, and VI had a minimum at the esterification time of 30 min. When combining all data of the incubation times of the esterification, the peak area ratios (*y*) of all peaks depended linearly upon the Cit concentration (in µM) (*x*): *y* = −0.223 + 0.011 *x*, *r*^2^ = 0.9943 for Peak I, *y* = 0.241 + 0.0098 *x*, *r*^2^ = 0.9712 for Peak II, *y* = −0.469 + 0.012 *x*, *r*^2^ = 0.9944 for Peak V, and *y* = −0.415 +0.013 *x*, *r*^2^ = 0.9923 for Peak VI. Linear relationships were observed between the peak area ratio (PAR) values (*y*) of the individual peaks and the derivatized Cit concentration (*x*) resulted in straight lines for all derivatization times of the esterification step (Figure 4). Linear regression analysis of the mean PAR of all peaks vs. the concentrations of derivatized Cit resulted in the regression equation *y* = −0.217 + 0.0117 *x*, *r*^2^ = 0.9973. The reciprocal of the slope value of this regression equation indicates a mean concentration of 85.3 µM for the sum of internal standards (nominal concentration, 100 µM).

Taken together, these results demonstrate the principle applicability of the procedure B for the quantitative GC-MS analysis of Cit in aqueous solutions.

### 3.3. Derivatization of Homocitrulline and Structural Characterization of Its Reaction Products by GC-MS

Scanning of the hCit samples derivatized by procedure B resulted in the elution each of an intense GC-MS peak using CH_3_OH (Figure S1D) and CD_3_OD (Figure S1E) and two minor peaks (Table 2).

The major GC-MS peaks eluted at 9.54 min and 9.52 min, respectively. The mass spectra of these peaks are very similar to those obtained from the derivatization of hCit by procedure B, as well as to those of the d_0_Me-PFP and d_3_Me-PFP derivatives of synthetic Lys confirming previous observations of the conversion of hCit to Lys [7] (Figure 1). The minor GC-MS peaks eluting at 9.21 min and 9.18 min could correspond to (*S*)-3-amino-2-oxoazepane-1-Carboxylic acid (Figure S1(D1,E1)). The minor GC-MS peaks eluting at 11.47 min and 11.42 min could correspond to *N*^6^-Carboxy-lysine (Figure S1(D3,E3)), analogous to *N*^5^-Carboxy-ornithine obtained from Cit using procedure B. The small differences in the retention times is indicative of the presence of deuterium atoms in the earlier eluting peaks.

## 4. Discussion

**Figure 5:**
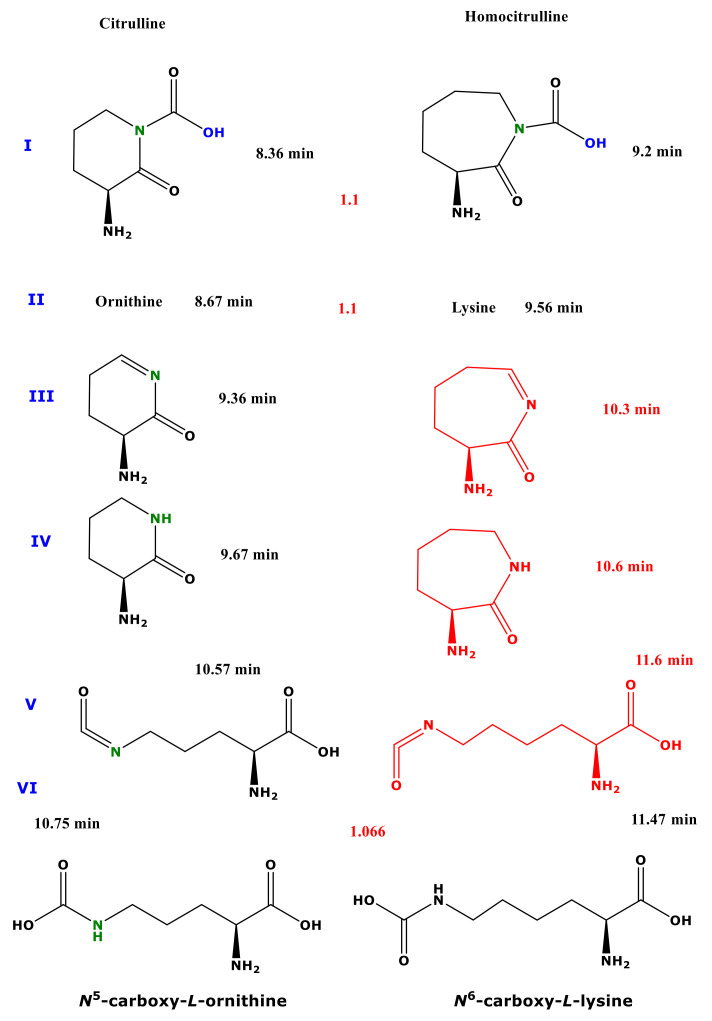
Proposed structures of the reaction products of citrulline (left panel) and homocitrulline (right panel) using procedure B (first PFPA/EA then 2 M HCl/MeOH). The red-marked structures were not found to be derivatization products of homocitrulline. The red numbers between the structures are the relative retention times of the homocitrulline products to the corresponding citrulline reaction products.

Procedure A allows for the reliable quantitative determination of amino acids and their metabolites in biological samples by GC-MS [7,10]. During the first esterification step, however, Cit and hCit undergo almost complete conversion to the methyl esters of Orn and Lys, respectively. The same happens to Gln and Asn, which are converted to the methyl esters of Glu and Asp, respectively [7]. These observations strongly indicate that the carbamide groups of Cit, hCit, Gln, and Asn are labile under the strong esterification conditions. This circumstance prevents simultaneous measurement of Cit, Orn, hCit, Lys, Gln, Glu, Asn, and Asp [7]. We have hypothesized that reversing the order of the derivatization procedure A may present a way to prevent the abovementioned conversions. In the present study, we investigated this possibility for Cit and hCit using procedure B, i.e., first pentafluoropropionylation and subsequently esterification, using previously optimized derivatization conditions [7]. Cit and hCit reacted to form five and three reaction products, respectively. The tentative chemical structures of these reaction products are illustrated in Figure 5.

One major reaction product of Cit was identified as Orn. This observation suggests that pentafluoropropionylation prevents conversion of Cit to Orn, albeit not entirely. The major reaction product of hCit was identified as Lys. The reaction products of hCit corresponding to the Cit-derived peaks III, IV, and V were not observed (Figure 5). These observations suggest that pentafluoropropionylation prevents conversion of hCit to Lys to only a minor extent. The conversion of Cit to four reaction products in addition to Orn suggest that pentafluoropropionylation of Cit enables additional reactions during the second reaction step of procedure B. The different reaction behaviour of Cit and hCit could be due to the longer side chain of these homologue amino acids: 3 vs. 4 CH_2_ groups. It is assumed that this structural difference plays a major role in the formation of cyclic reaction products (Figure 5). Interestingly, procedure B resulted in the formation of *N*^5^-Carboxy-Orn from Cit as a major reaction product and *N*^6^-Carboxy-Lys from hCit as a minor reaction product. Because of the commercially unavailability of synthetic standards of *N*^5^-Carboxy-*L*-Orn and *N*^6^-Carboxy-*L*-Lys, we were not able to unequivocally demonstrate the formation of these reaction products. Nevertheless, these putative reaction products enable discrimination of Cit from Orn, and of hCit from Lys, respectively. It is interesting to note that the physiological occurrence and the biological significance of the free amino acids *N*^5^-Carboxy-Orn and *N*^6^-Carboxy-Lys (Chemical Entities of Biological Interest (ChEBI):43575) have not been reported thus far. However, a *N*^6^-Carboxy-Lys residue was found to be present in the active site of class D β-lactamases and to play a significant role in the hydrolysis of β-lactam antibiotics [11,12]. Our study provides useful information for forthcoming studies on these uncommon amino acids.

**Figure 6:**
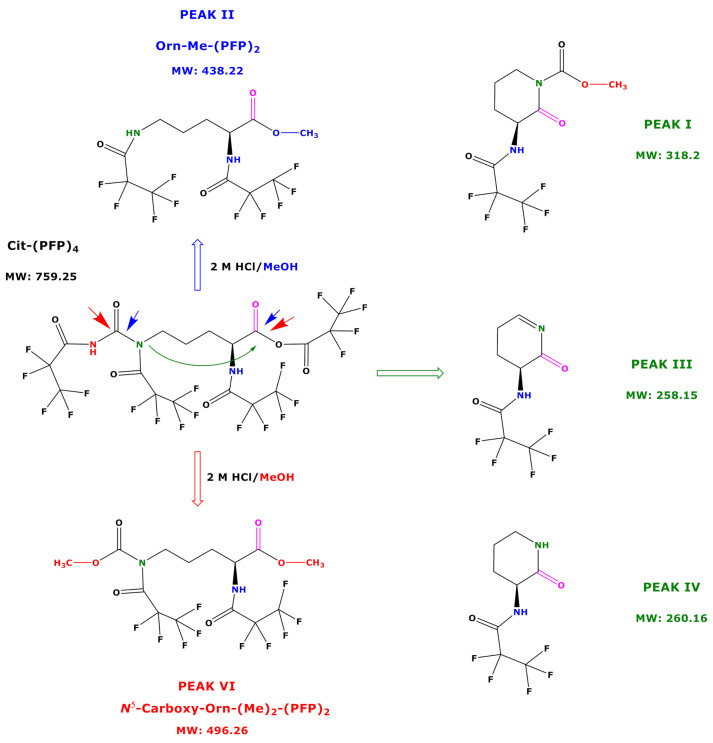
Proposed reaction products of the very first, not yet identified *N*,*N*,*N*,*O*-(PFP)_4_ derivative of Cit using procedure B. Blue and red arrows indicate the carbonyl moieties, which are attacked by methanol, and the green arrow indicates the intramolecular attack of *N*^5^ on *C*^1^ carbonyl group.

Based on the results of our study, we propose potential mechanisms that may explain the reaction products of Cit and hCit during the derivatization procedure B. Being a highly reactive derivatization reagent, PFPA is likely to react with all functional groups of free amino acids and those in tripeptides [8,9]. We therefore assume that PFPA/EA reacts with all functional groups of Cit to form its *N*,*N*,*N*,*O*-(PFP)_4_ derivative (Figure 6). An intact Cit-(PFP)_4_ derivative was not observed in our study. An explanation could be that the remaining Cit-(PFP)_4_ extracted into toluene decomposed during the injection in the hot injector (280 °C). This is more likely to happen to the *O*-PFP residue, as *N*-PFP residues of derivatized amino acids are considerably stable [7]. A more plausible explanation for our observations is that the *O*-PFP residue of the Cit derivative is a mixed anhydride of PFPA and the carboxylic group Cit. As such, the Cit-(PFP)_4_ derivative is likely to undergo several reactions with 2 M HCl/CH_3_OH (Figure 6). The reaction of the Cit-(PFP)_4_ derivative with 2 M HCl/CH_3_OH will always generate its *C*^1^-Carboxy-methyl ester. Analogously, the reaction of the Cit-(PFP)_4_ derivative with 2 M HCl/CD_3_OD will generate the *C*^1^-Carboxy-trideutero-methyl ester. This provides a way to prepare deuterium-labelled internal standards for quantitative analyses. Especially the *N*-PFP residue on the carbamide functionality of the Cit-(PFP)_4_ derivative opens ways for additional reactions, which leads to the formation of open reaction products including *N*^5^-Carboxy-Orn from Cit and *N*^6^-Carboxy-Lys from hCit and several cyclic reaction products that can be utilized both in analytical and organic preparative chemistry (Figure 6).

The reaction time of the esterification reaction performed at 80 °C has an effect on the yield of individual reaction products. In a proof-of-principle experiment, we found that procedure B is useful for the quantitative analysis of Cit in aqueous solution for several esterification times. Yet, the quantitative determination of Cit, Orn, hCit, and Lys in biological samples by GC-MS using procedure B remains to be optimized and validated. Our preliminary studies suggest that the derivatization procedure B can be extended to Gln and Asn, which are converted into Glu and Asp, respectively. The derivatization procedure B possess the potential to simultaneously quantitate a large number of biological amino acids and their metabolites by GC-MS using in situ prepared (d_3_Me)_m_-(PFP)_n_ or commercially available stable-isotope labelled amino acids as internal standards.

## Figures and Tables

**Table 1:** GC-MS retention times (*t*_R_, min) and most intense ions in the mass spectra of the six reaction products of citrulline derivatized with procedure B (first PFPA/EA then 2 M HCl/CH_3_OH or 2 M HCl/CD_3_OD). For comparison, synthetic ornithine standard (Orn-Std) was also derivatized with procedure B. See also Figure S1.

Peak No.	*t*_R_	Spectrum Intensity	*m/z* (Intensity, %)
Peak I	8.36	1.0 × 10^7^	235 (2), 278 (5), **298** ^a^ (100), 318 (2)
Peak I	8.36	1.3 × 10^7^	238 (2), 281 (4), **301** (100), 321 (1)
Peak II	8.67	1.5 × 10^7^	275 (5), 398 (18), **418** (100)
Peak II	8.65	1.2 × 10^7^	278 (5), 401 (20), **421** (100)
Peak III	9.36	1.2 × 10^6^	218 (92), **238** (100)
Peak III	9.36	1.5 × 10^6^	218 (90), **238** (100)
Peak IV	9.68	2.8 × 10^6^	220 (20), **240** (100), 258 (5)
Peak IV	9.67	3.0 × 10^6^	220 (15), **240** (100), 258 (5)
Peak V	10.57	2.5 × 10^6^	162 (43), 278 (15), **298** (100), 486 (2)
Peak V	10.52	2.7 × 10^6^	162 (25), 281 (12), **301** (100), 489 (6)
Peak VI	10.75	2.5 × 10^7^	290 (5), 298 (27), **330** (100), 349 (3)
Peak VI	10.71	2.9 × 10^7^	296 (5), 301 (25), **336** (100), 355 (3)
Orn-Std	8.67	1.1 × 10^8^	275 (3), 398 (20), **418** (100), 437 (8)
Orn-Std	8.63	1.2 × 10^8^	278 (2), 401 (20), **421** (100), 440 (10)

^a^ Bold numbers indicate mass fragments with the highest intensity in the mass spectrum (i.e., base peaks).

**Table 2:** GC-MS retention times (*t*_R_, min) and most intense ions in the mass spectra of the three reaction products of homocitrulline derivatized with procedure B (PFPA/EA then 2 M HCl/CH_3_OH or 2 M HCl/CD_3_OD). For comparison, synthetic lysine standard (Lys-Std) was also derivatized with procedure B. See also Figure S1.

Peak No.	*t*_R_	Spectrum Intensity	*m/z* (Intensity, %)
Peak I	9.21	4.7 × 10^5^	272 (9), 292 (5), **312** ^a^ (100), 338 (3)
Peak I	9.18	5.9 × 10^5^	272 (8), 295 (3), **315** (100), 338 (3)
Peak II	9.54	2.5 × 10^7^	289 (11), 392 (8), 412 (27), **432** (100)
Peak II	9.52	1.1 × 10^7^	292 (10), 395 (5), 415 (27), **435** (100)
Peak III	11.47	4.0 × 10^5^	**312** (100), 344 (42)
Peak III	11.42	7.7 × 10^5^	**315** (100), 350 (48)
Lys-Std	9.54	5.6 × 10^6^	289 (15), 392 (16), 412 (50), **432** (100), 451 (5)
Lys-Std	9.52	5.6 × 10^6^	292 (16), 395 (14), 415 (30), **435** (100), 454 (4)

^a^ Bold numbers indicate mass fragments with the highest intensity in the mass spectrum (i.e., base peaks).

## Data Availability

The study did not report any data.

## Supplementary Materials

The following are available online, Figure S1: Separate derivatization of citrulline and homocitrulline (5 nmol each) by procedure B, i.e., first with PFPA/EA and then 2 M HCl/CH_3_OH or 2 M HCl/CD_3_OD, and structural characterization of their reaction products by GC-MS.

## Sample Availability

Samples of the compounds are not available from the authors.
