# Supplementary material for: GC-MS Discrimination of Citrulline from Ornithine and Homocitrulline from Lysine by Chemical Derivatization: Evidence of Formation of N5-Carboxy-ornithine and N6-Carboxy-lysine
Source: Molecules. 2021 Apr 15;26(8):2301. doi: 10.3390/molecules26082301 (PMC8071523; doi:10.3390/molecules26082301)

Article

GC-MS Discrimination of Citrulline from Ornithine and Homocitrulline from Lysine by Chemical Derivatization: Evidence of Formation of *N*⁵-Carboxy-ornithine and *N*⁶-Carboxy-lysine

Svetlana Baskal, Alexander Bollenbach and Dimitrios Tsikas *

Institute of Toxicology, Core Unit Proteomics, Hannover Medical School, *Hannover* 30625, Germany; baskal.svetlana@mh-hannover.de (S.B.); bollenbach.alex@gmail.com (A.B.)

* Correspondence: Tsikas.dimitros@mh-hannover.de

Citation: Baskal, S.; Bollenbach, A.; Tsikas, D. GC-MS Discrimination of Citrulline from Ornithine and Homocitrulline from Lysine by Chemical Derivatization: Evidence of Formation of *N*⁵-Carboxy-ornithine and *N*⁶-Carboxy-lysine. *Molecules* **2021**, *26*, 2301. https://doi.org/10.3390/molecules26082301

Academic Editor: Paraskevas D. Tzanavaras

Received: 24 March 2021

Accepted: 13 April 2021

Published: date

Publisher's Note: MDPI stays neutral with regard to jurisdictional claims in published maps and institutional affiliations.

Copyright: © 2021 by the authors. Licensee MDPI, Basel, Switzerland. This article is an open access article distributed under the terms and conditions of the Creative Commons Attribution (CC BY) license (http://creativecommons.org/licenses/by/4.0/).

Figure S1. Separate derivatization of citrulline and homocitrulline (5 nmol each) by procedure B, i.e., first with PFPA/EA and then 2 M HCl/CH_3OH or 2 M HCl/CD_3OD, and structural characterization of their reaction products by GC-MS.

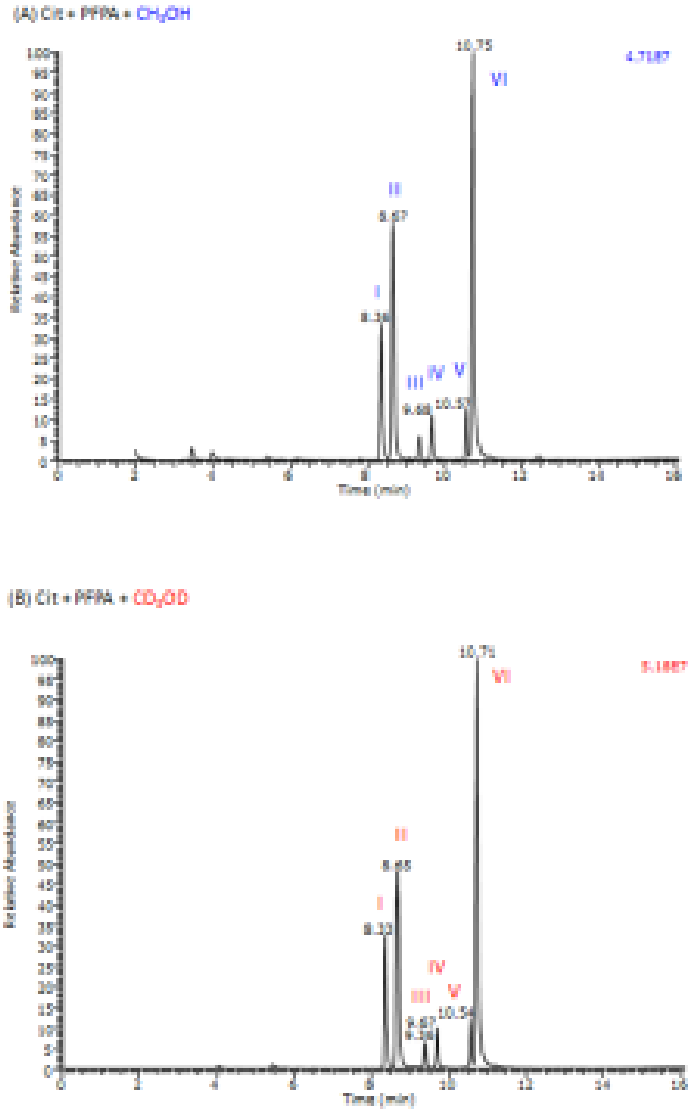

(A1) Cit + PFPA + CH_3OH: Peak I, t_R=8.36 min

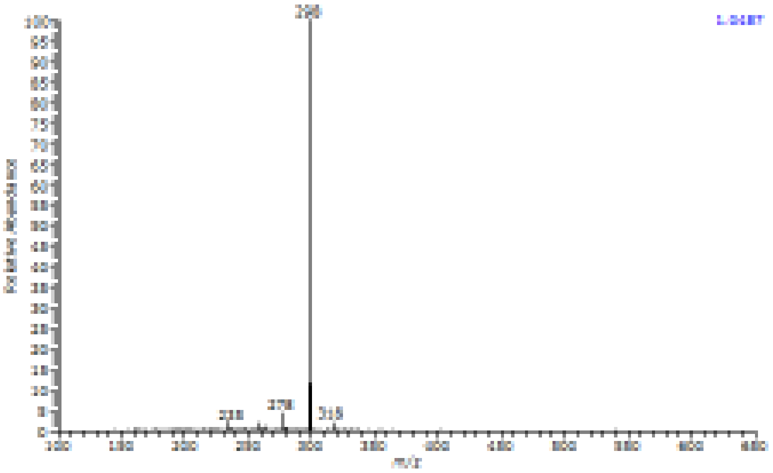

Peak I

tR 8.36/8.33

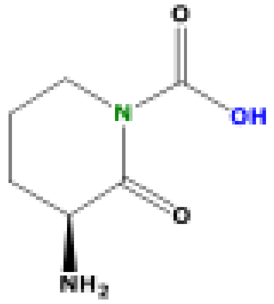

(B1) Cit + PFPA + CD_3OD: Peak I, t_R=8.33 min

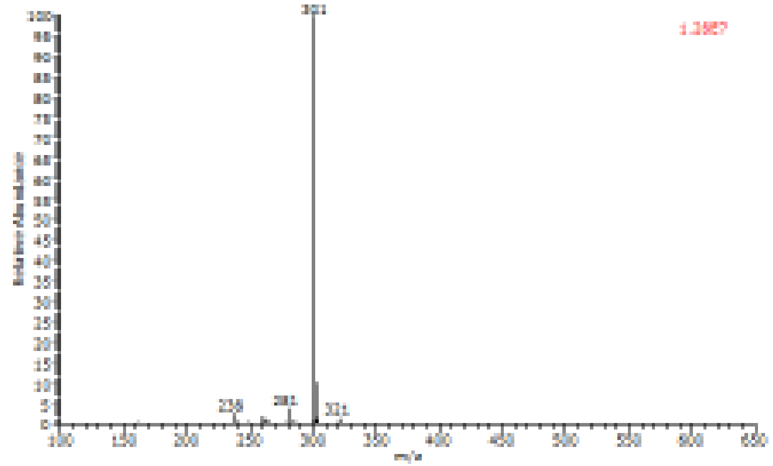

(A2) Cit + PFPA + CH_3OH: Peak II, t_R=8.67 min

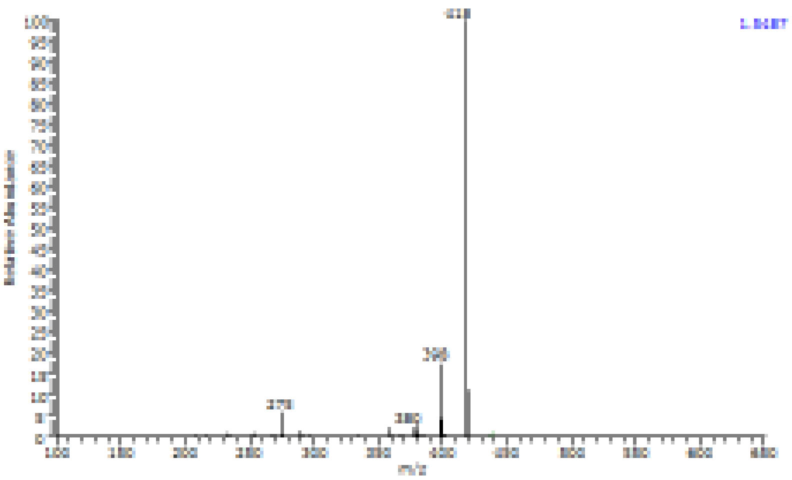

(B2) Cit + PFPA + CD_3OD: Peak II, t_R=8.63 min

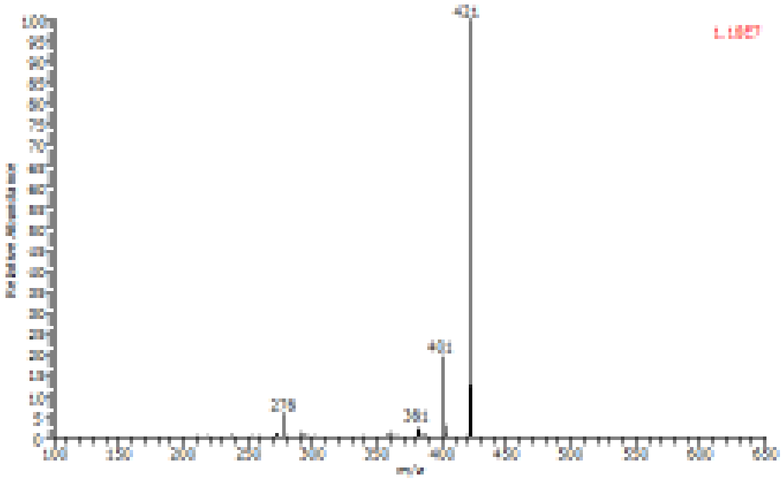

Peak II

Ornithine from Cit

tR 8.67/8.63

(A3) Cit + PFPA + CH_3OH: Peak III, t_R=9.36 min

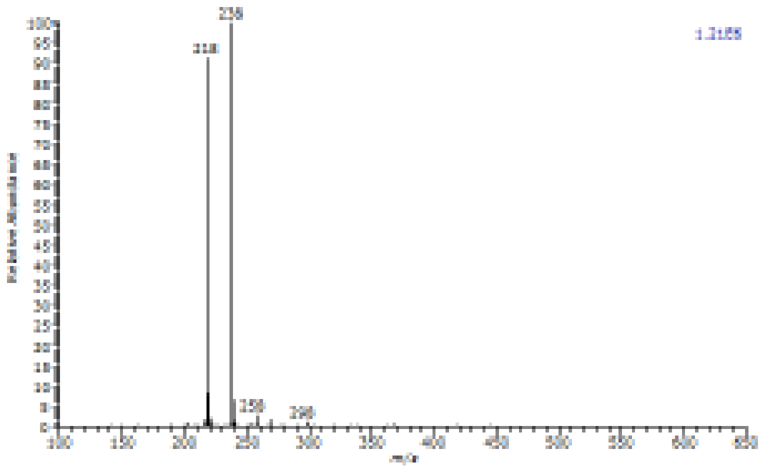

Peak III

tR 9.36/9.36

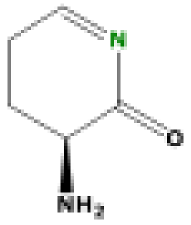

(B3) Cit + PFPA + CD_3OD: Peak III, t_R=9.36 min

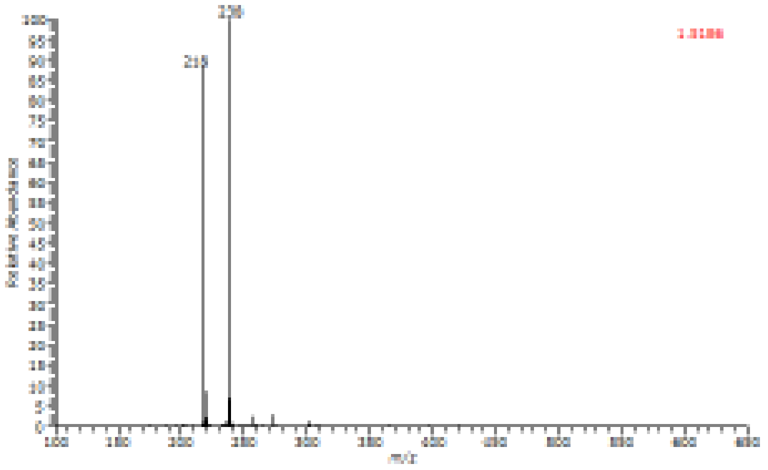

(A4) Cit + PFPA + CH_3OH: Peak IV, t_R=9.67 min

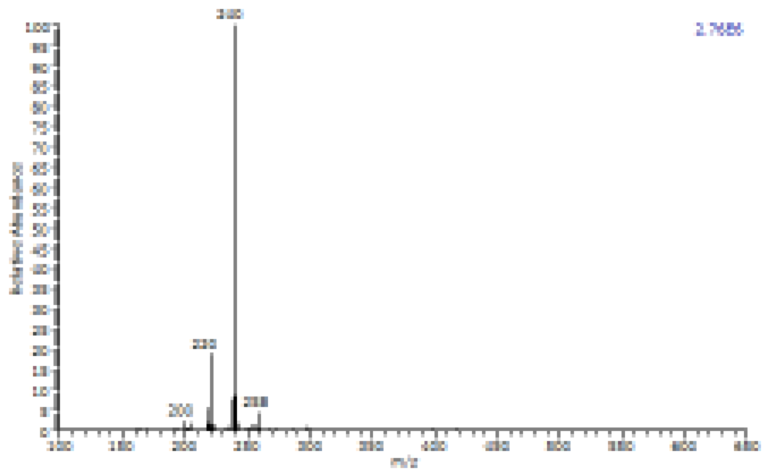

Peak IV

tR 9.67/9.63

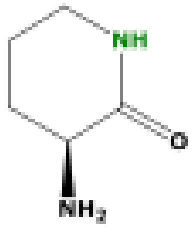

(B4) Cit + PFPA + CD_3OD: Peak IV, t_R=9.38 min

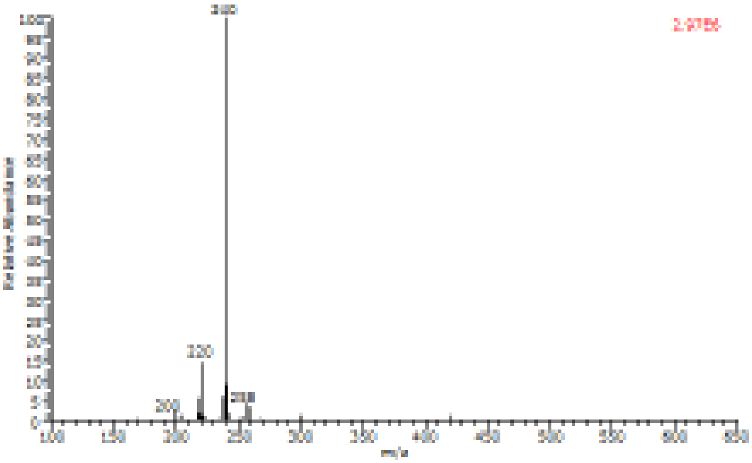

(A5) Cit + PFPA + CH_3OH: Peak V, t_R=10.57 min

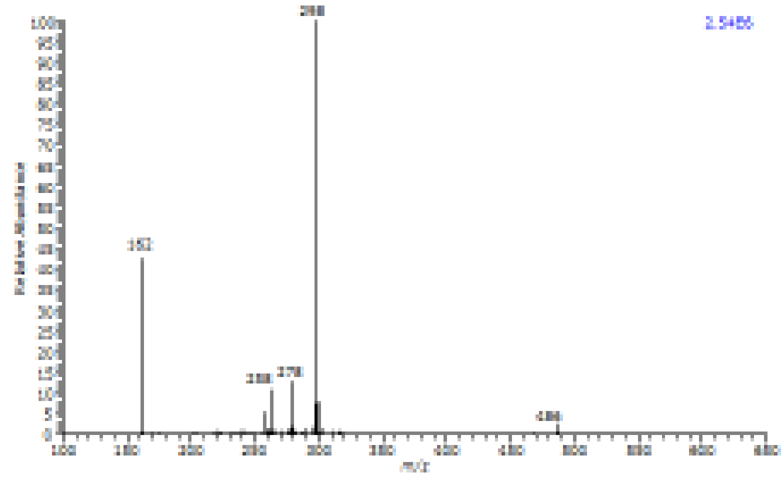

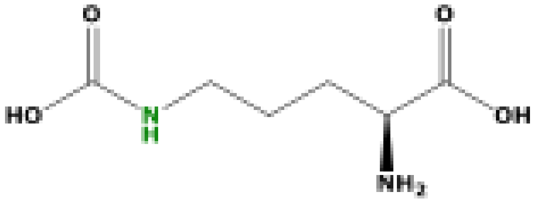

tR 10.57/10.52

from Cit

N^{5}-Carboxy-Ornithin

(B5) Cit + PFPA + CD_3OD: Peak V, t_R=10.54 min

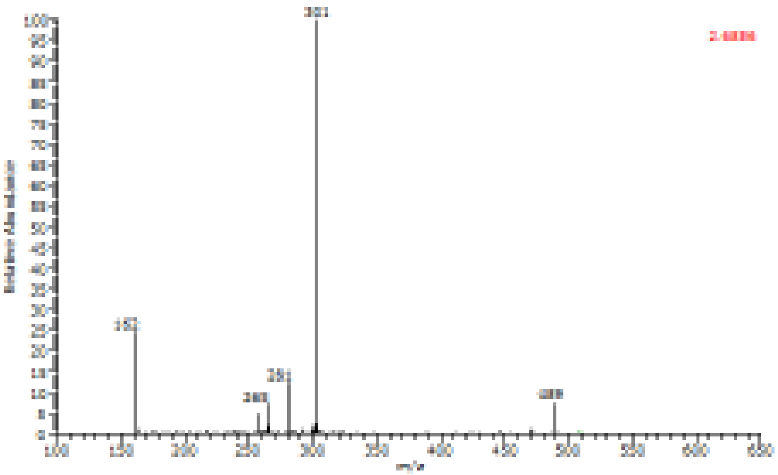

(A6) Cit + PFPA + CH_3OH: Peak VI, t_R=10.75 min

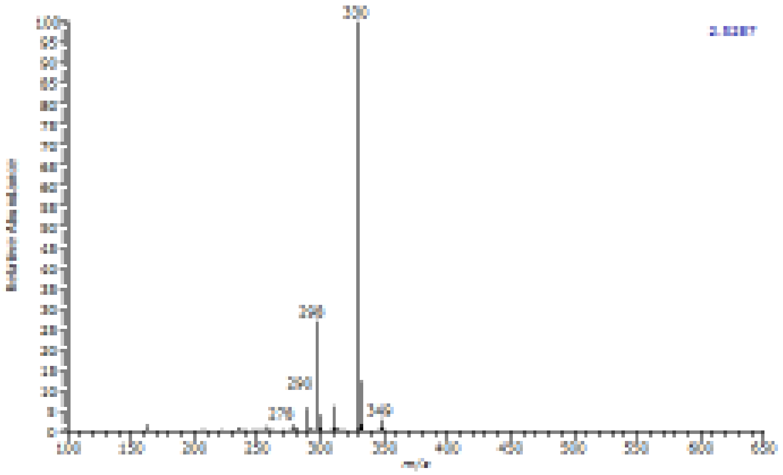

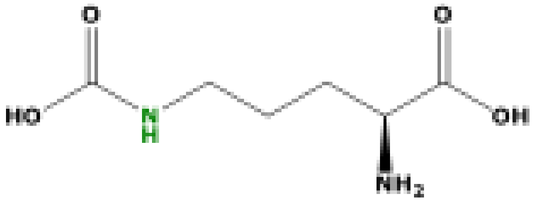

Peak VI

tR 10.75/10.71

(B6) Cit + PFPA + CD_3OD: Peak VI, t_R=10.71 min

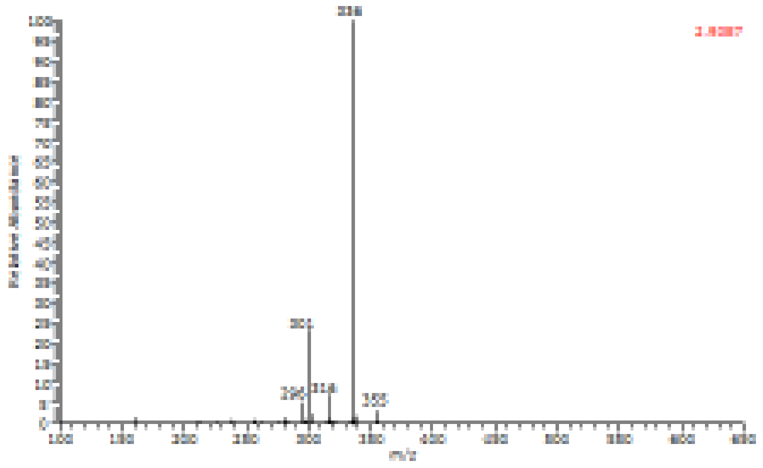

(D) hCit+ PFPA + CH_3OH

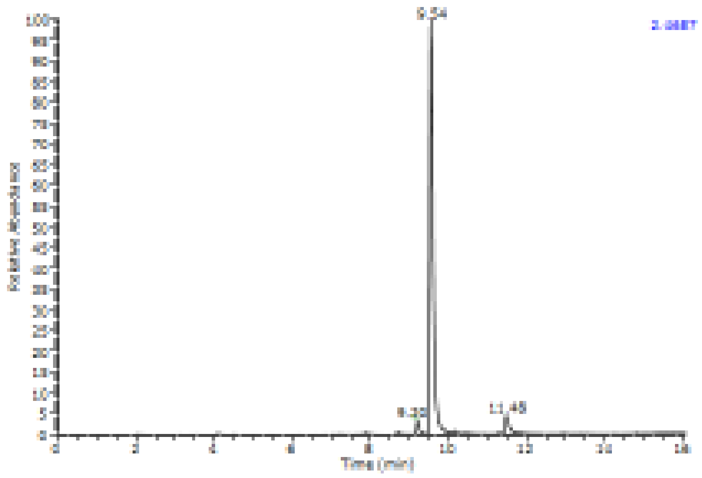

tR 9.54/9.52

Lysine

from hCit

(E) hCit+ PFPA + CD_3OD

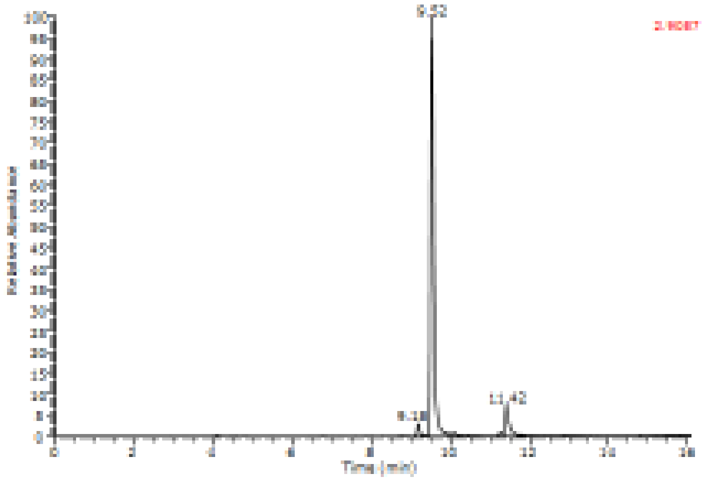

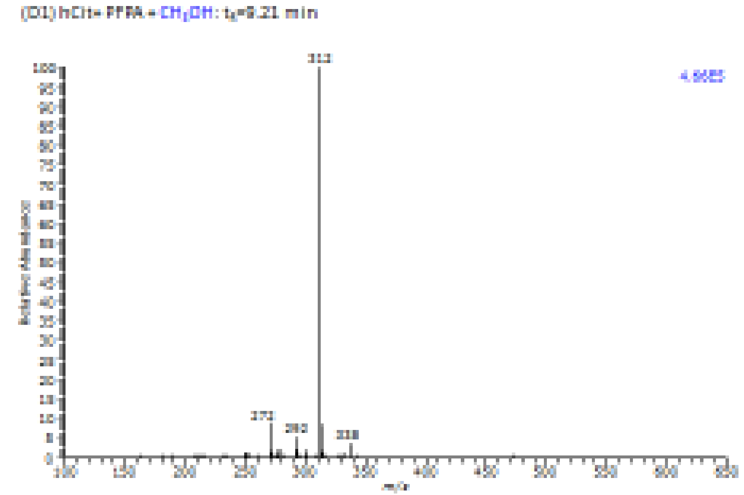

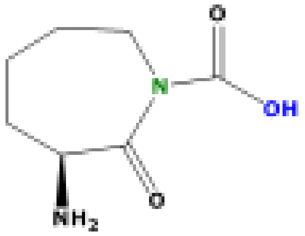

tR 9.21/9.18

from hCit

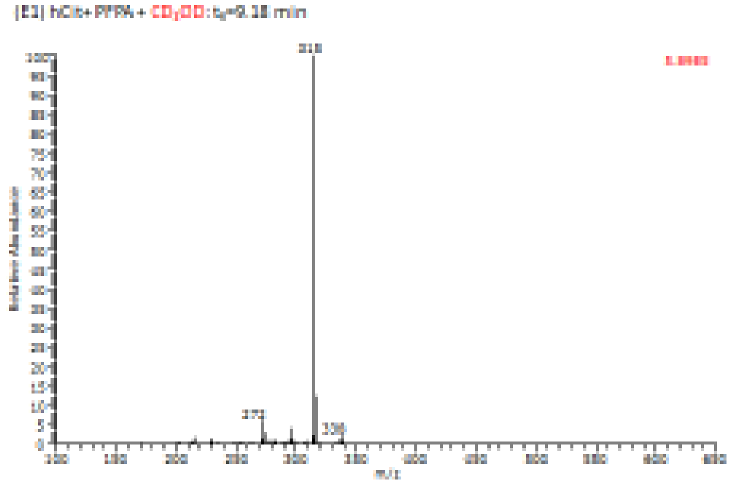

(D3) hCit+ PFPA + CH_3OH: t_R=11.47 min

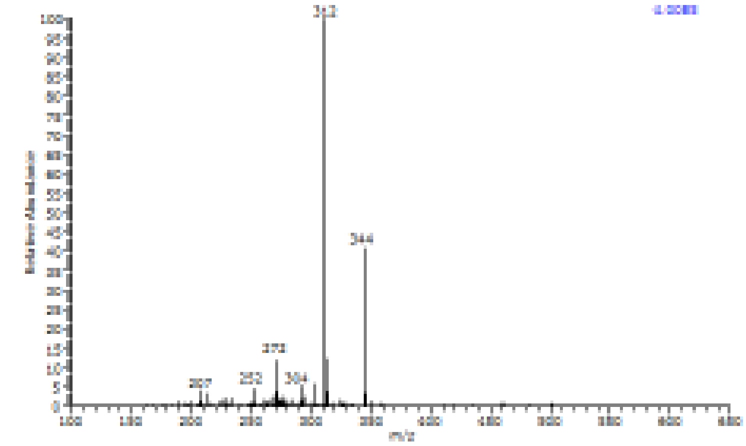

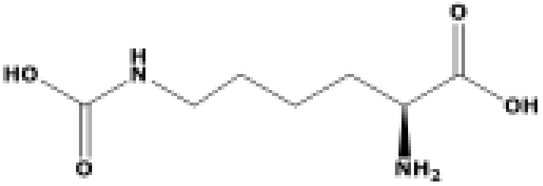

tR 11.47/11.42

from hCit

***N*[6]-carboxy-*L*-lysine**

(E3) hCit+ PFPA + CD_3OD: t_R=11.42 min

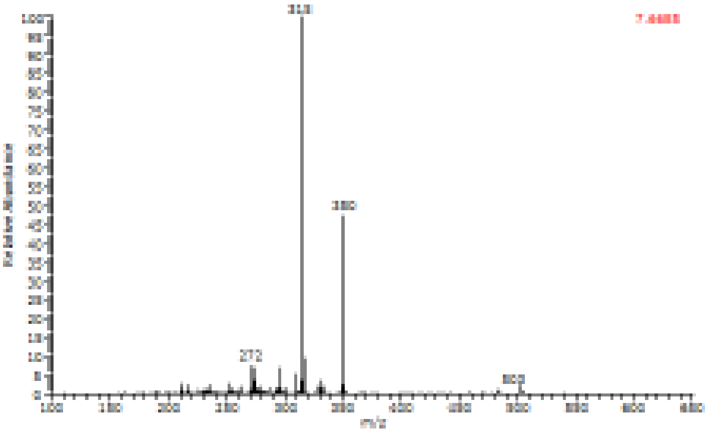

(D3) hCit+ PFPA + CH_3OH: t_R=11.47 min

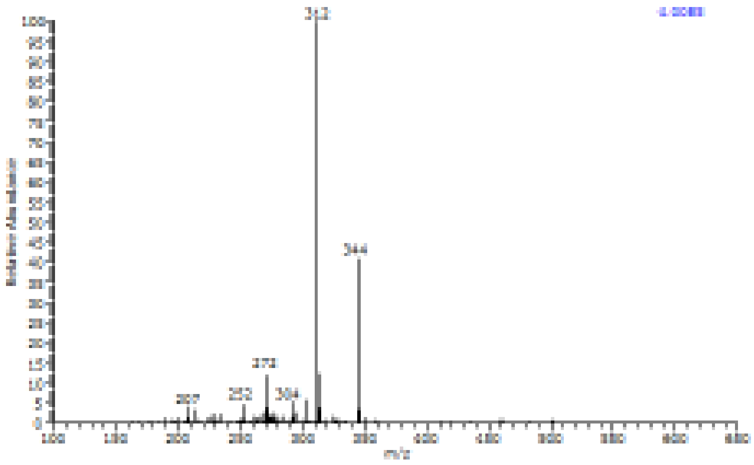

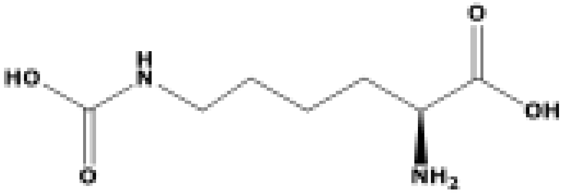

tR 11.47/11.42

from hCit

***N*[6]-carboxy-*L*-lysine**

(E3) hCit+ PFPA + CD_3OD: t_R=11.42 min

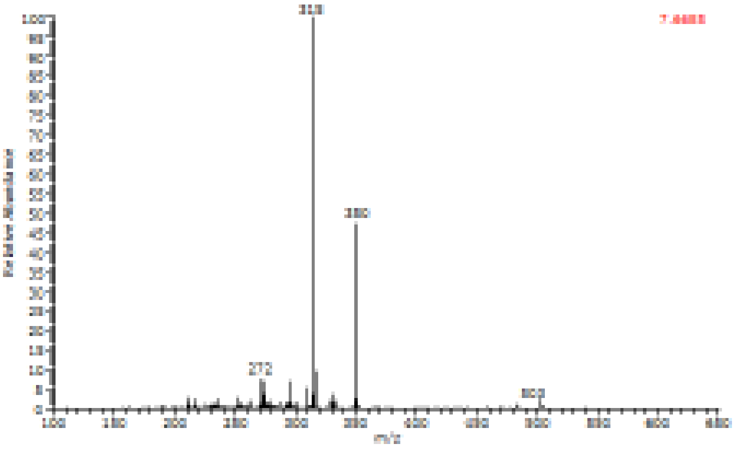

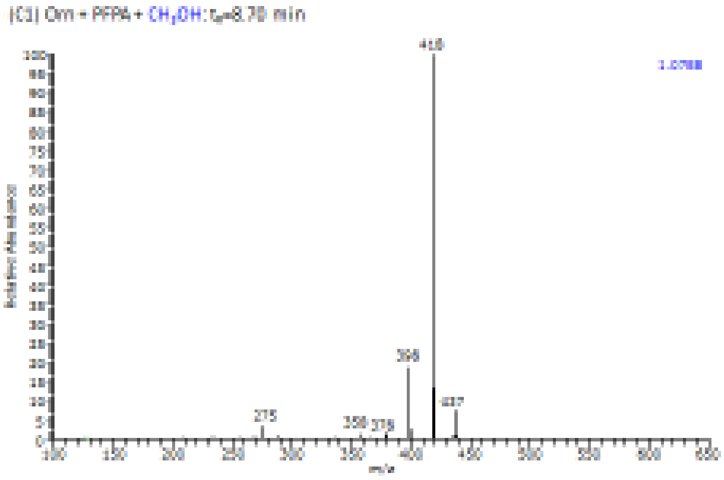

Ornithine-Standard

tR 8.67/8.63

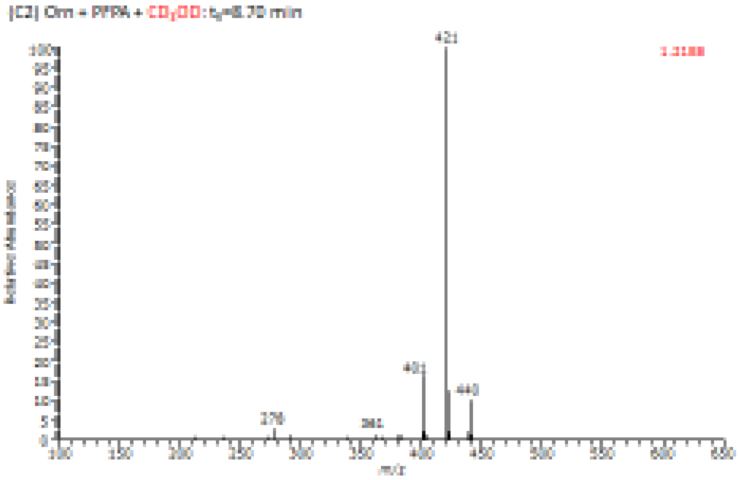

Supplement: Supplementary file 1 [file molecules-26-02301-s001.zip › molecules-1176304-supplementary.pdf]
